# Acquired Hemophilia A in IgG4-Related Disease: Case Report, Immunopathogenic Study, and Review of the Literature

**DOI:** 10.3389/fimmu.2020.558811

**Published:** 2020-12-18

**Authors:** Sébastien Sanges, Emmanuelle Jeanpierre, Benjamin Lopez, Jules Russick, Sandrine Delignat, Benjamin Carpentier, Romain Dubois, Sylvain Dubucquoi, Thomas Guerrier, Éric Hachulla, Pierre-Yves Hatron, Camille Paris, Sophie Susen, David Launay, Sébastien Lacroix-Desmazes, Louis Terriou

**Affiliations:** ^1^ Univ. Lille, U1286-INFINITE-Institute for Translational Research in Inflammation, Lille, France; ^2^ Inserm, Lille, France; ^3^ CHU Lille, Département de Médecine Interne et Immunologie Clinique, Lille, France; ^4^ CHU Lille, Département de Médecine Interne et Immunologie Clinique, Centre de Référence des Maladies Auto-Immunes Systémiques Rares du Nord et Nord-Ouest, Lille, France; ^5^ Health Care Provider of the European Reference Network on Rare Connective Tissue and Musculoskeletal Diseases Network (ReCONNET), Lille, France; ^6^ CHU Lille, Institut d’Hématologie Transfusion, Lille, France; ^7^ INSERM, U1011, Univ. Lille, U1011-EGID, Institut Pasteur de Lille, Lille, France; ^8^ CHU Lille, Institut d’Immunologie, Lille, France; ^9^ Centre de recherche des Cordeliers, INSERM, Sorbonne Université, Université de Paris, Paris, France; ^10^ Service d’Hématologie, Hôpital Saint-Vincent, GHICL, Lille, France; ^11^ CHU Lille, Institut de Pathologie, Lille, France

**Keywords:** anti-factor VIII autoantibodies, IgG4 antibodies, acquired hemophilia A, IgG4-related disease, plasma cell

## Abstract

We report the observation of a 75-year-old patient referred for cervical lymphadenopathies. A pre-lymphadenectomy blood work revealed an asymptomatic elevation of aPTT with low factor VIII (FVIII) levels and high anti-FVIII antibodies titers, consistent with acquired hemophilia A (AHA). Histological work-up of a cervical lymphadenopathy revealed benign follicular hyperplasia with IgG4^+^ lymphoplasmacytic infiltration; and serum IgG4 levels were markedly elevated, compatible with IgG4-related disease (IgG4-RD). He was successfully treated with a 9-month course of prednisone, secondarily associated with rituximab when an AHA relapse occurred. As this patient presented with an unusual association of rare diseases, we wondered whether there was a link between the two conditions. Our first hypothesis was that the anti-FVIII autoantibodies could be directly produced by the proliferating IgG4^+^ plasma cells as a result of broken tolerance to autologous FVIII. To test this assumption, we determined the anti-FVIII IgG subclasses in our patient and in a control group of 11 AHA patients without IgG4-RD. The FVIII inhibitor was mostly IgG4, with an anti-FVIII IgG4/IgG1 ratio of 42 at diagnosis and 268 at relapse in our patient; similar values were observed in non-IgG4-RD AHA patients. As a second hypothesis, we considered whether the anti-FVIII activity could be the result of a non-specific autoantibody production due to polyclonal IgG4^+^ plasma cell proliferation. To test this hypothesis, we measured the anti-FVIII IgG4/total IgG4 ratio in our patient, as well as in several control groups: 11 AHA patients without IgG4-RD, 8 IgG4-RD patients without AHA, and 11 healthy controls. We found that the median [min-max] ratio was higher in AHA-only controls (2.4 10^-2^ [5.7 10^-4^-1.79 10^-1^]), an oligoclonal setting in which only anti-FVIII plasma cells proliferate, than in IgG4-RD-only controls (3.0 10^-5^ [2.0 10^-5^-6.0 10^-5^]), a polyclonal setting in which all IgG4^+^ plasma cells proliferate equally. Our patient had intermediate ratio values (2.7 10^-3^ at diagnosis and 1.0 10^-3^ at relapse), which could plead for a combination of both mechanisms. Although no definitive conclusion can be drawn, we hypothesized that the anti-FVIII autoantibody production in our IgG4-RD AHA patient could be the result of both broken tolerance to FVIII and bystander polyclonal IgG4^+^ plasma cell proliferation.

## Introduction

Immunoglobulin G4 (IgG4)-related disease (IgG4-RD) is a rare immune-mediated condition characterized by elevated serum IgG4 levels and IgG4^+^ lymphoplasmacytic infiltration of the involved organs (1). Although it is associated with various auto-immune disorders (some of which being autoantibody-mediated), it is unclear whether the produced IgG4 are directly pathogenic (2). The involved plasma cell population is historically thought to be polyclonal in nature, but some have suggested that the response could be oligoclonal and compatible with an antigen-driven immune response (1).

Acquired hemophilia A (AHA) is a rare and severe bleeding disorder caused by the development of autoantibodies against coagulation factor VIII (FVIII). AHA can be idiopathic or associated with other diseases, including immunologic disorders (mainly rheumatoid arthritis and systemic lupus erythematosus) (3). Occurrence of AHA in IgG4-RD has exceptionally been reported (4–6), and only one study has explored a possible immunopathogenic link between the 2 diseases (4).

Here, we report the case of a patient presenting with this association, in whom we investigated whether the anti-FVIII autoantibody production was related to the IgG4^+^ plasma cell proliferation.

## Methods

### Patient and Controls

In order to study the immunologic associations between AHA and IgG4-RD, results obtained in our patient were compared to three control groups. The first control group consisted of 11 patients with a diagnosis of AHA (based on low FVIII plasma levels and detectable anti-FVIII activity) and no evidence of IgG4-RD (i.e.,normal IgG4 serum levels and no sign of organ involvement on clinical examination and on chest and abdominal CT scan). The second control group consisted of 8 patients with a diagnosis of IgG4-RD [based on the 2019 ACR/EULAR classification criteria (7)] and no evidence of AHA (i.e., normal aPTT). The third control group consisted of 11 healthy blood donors.

Relevant clinical and biological data were retrospectively collected from medical records. Additional analyses needed for this study were performed on remaining blood samples initially collected for patient care.

### Measurement of Plasma FVIII Levels and Anti-FVIII Activity

Plasma FVIII levels were determined by one-stage clot assay, using Triniclot APTT-HS (T Coag, Stago) and FVIII-deficient plasma (Siemens, Marburg, Germany) on Sysmex CS-2400 (Sysmex, Japan). The Bethesda assay was used to determine inhibitor potency, with inhibitor concentrations ≥ 0.6 BU defined as positive. We used the Nijmegen-modified Bethesda assay (8, 9) (buffering the normal pool plasma substrate with imidazole to pH 7.4 and using imidazole buffer in the control incubation mix). In brief, serial dilutions of plasma (imidazole buffer) were incubated with an equal volume of normal pooled plasma (NPP) (Standard Human Plasma, Siemens, Marburg, Germany) at 37°C for 2 h. FVIII:C activity was determined by one‐stage clot assay described above. The residual activity (RA) was determined as a percentage of the activity of a control mixture of NPP with imidazole buffer 1:1. The inhibitor titer in Bethesda units represented the reciprocal of the dilution of the patient’s plasma that produced 50% inhibition of FVIII.

### Measurement of Total IgG and IgG Subclasses Concentrations

Total IgG and IgG subclass measurements were performed on patient sera according to the manufacturer’s instructions using commercially available Optilite^®^ assays (references: NK004.OPT, NK006.OPT, NK007.OPT, LK008.OPT and LK009.OPT for total IgG and IgG1/2/3/4 subclasses, respectively), on the fully automated Optilite^®^ turbidimetric analyzer (The Binding Site Group Ltd, Birmingham, UK). Each assay comprised a specific antiserum for the targeted IgG subclass, and specific calibrators and controls that have been calibrated against the ERMDA470k certified reference material.

### Measurement of Serum Anti-FVIII IgG, IgG1, and IgG4 levels

ELISA plates (Nunc, Roskilde, Denmark) were coated with recombinant FVIII (2 μg/ml, Advate^®^, Takeda, Vienna, Austria) overnight at 4°C. After blocking with PBS-3% bovine serum albumin (BSA), plasma diluted in PBS-3% BSA was incubated for 1 hr at 37°C. Bound IgG1, IgG4, or total IgG were revealed using HRP-conjugated monoclonal anti-human Fc IgG1 (clone HP6001, Southern Biotech Anaheim, CA, USA), IgG4 (clone HP6025, Southernbiotech) or IgG (clone JDC-10, Southern Biotech), respectively, and the OPD substrate (Sigma-Aldrich Chemie GmbH, Munich, Germany). As a standard, the recombinant anti-FVIII monoclonal IgG4 [BO2C11 clone (10)] and IgG1 [BOIIB2 clone (11)] were used.

### Immunostaining of Lymph Node Sections

In order to better assess T cell responses, lymph node sections from the axillary biopsy were stained using CD4 (clone SP35, Roche Ventana), CD8 (clone C8-144B, Dako), PD1 (clone NAT105, Roche Ventana) and FOXP3 (clone 236A/E7, Abcam).

## Results

### Case Description

A 75-year-old Caucasian male with no previous medical history was referred to our tertiary care center for prolonged aPTT.

Several months before referral, he reported bilateral parotid enlargement and cervical lymphadenopathies. Conventional face, neck, chest, and abdominal CT scan revealed cervical, mediastinal, and axillary lymphadenopathies with diffuse bilateral parotid enlargement. These lesions showed significant 18-flurodesoxyglucose avidity on positron emission tomography CT scan. An axillary lymph node biopsy was decided but postponed due to a prolonged aPTT discovered on preoperative laboratory tests.

On admission, the patient showed no sign of bleeding. There was no other significant anomaly on clinical examination. Biological work-up showed normal kidney, liver, pancreas, and thyroid functions. Acute phase reactants and immunological tests were within normal range.

**Figure 1:**
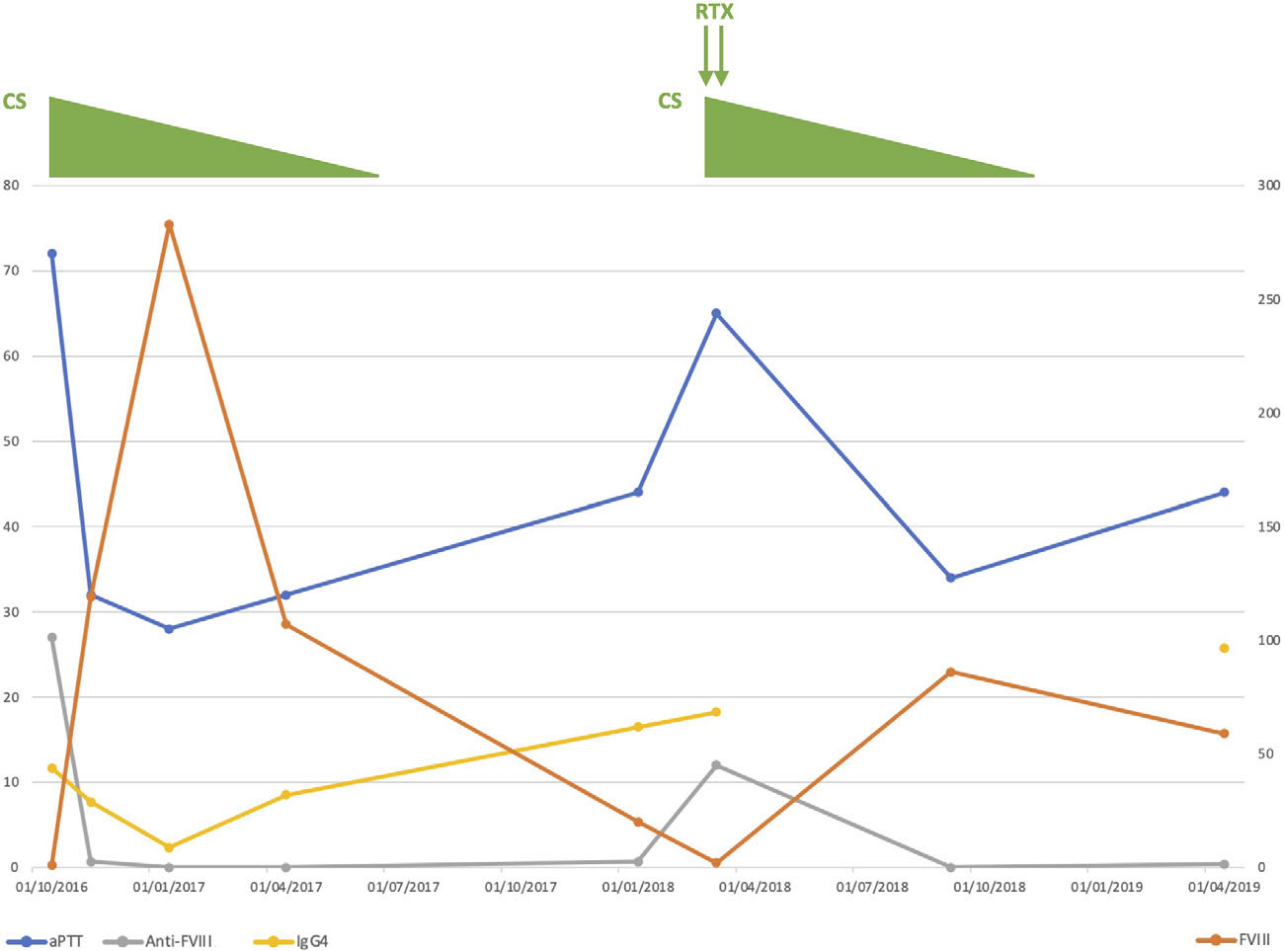
Clinical course of our patient. Anti-FVIII, anti-FVIII activity; aPTT, activated partial thromboplastin time; CS, corticosteroids; FVIII, coagulation factor VIII; Ig, immunoglobulin; RTX, rituximab.

We confirmed the prolonged aPTT with normal PT and found decreased FVIII plasma levels associated with positive anti-FVIII IgG4 autoantibodies (**Table 1** and **Figure 1**), leading to the diagnosis of AHA. No hemorrhagic complication was noted.

**Table 1:** Clinical and biological data of our patient and other IgG4-RD-associated AHA cases.

Case	Sex	Age	Delaybetween diseases	Timeframe	Characteristics of IgG4-RD			Characteristics of AHA				Treatment		
					Organinvolvements	IgG(g/l)	IgG4(g/l)	Bleeding sites	aPTT(s)	FVIII(%)	Anti-FVIII(BU/ml)	IS	Bypassingagents	Outcome
Our patient	M	75	AHA simultaneous to IgG4-RD	At AHA diagnosis	Parotid glands Lymph nodes		11.70	None	72	1	27	CS	Yes*	Remission at last follow-up (36 months from AHA diagnosis)
		75		At month 3			2.27	None	28	283	0	CS	No	
		76		At month 6			8.52	None	32	107	0	CS	No	
		77		At AHA relapse (month 18)			18.22	None	65	2	12	CS + RTX	No	
Narazaki et al. (4)	F	72	AHA 8y after IgG4-RD	At AHA diagnosis	Pancreas Submandibular glands	11.7	1.44	Subcutaneous and muscle hematomas, hematuria	80	<1	290	CS + CYC	Yes	Remission at last follow-up (21 months from AHA diagnosis)
				At AHA relapse (month 8)			Normal (≈0.10)	Subcutaneous hematoma	≈60	2	48	CS + FK	Yes	
Li et al. (5)	M	55	AHA simultaneous to IgG4-RD	At AHA diagnosis	Lung, biliary duct, pancreas, lymph nodes, urinary tract, parotid glands	21.4	>4.03	Subcutaneous hematoma	120	0.5	27.2	CS	No	Remission at last follow-up (4 months from AHA diagnosis)
Sugino et al. (6)	M	66	AHA 1y after IgG4-RD	At AHA diagnosis	Lung, biliary duct, pancreas	8.7	3.92	Muscle hematoma	58	2	20	CS	Yes	Remission at last follow-up (25 months from AHA diagnosis)

*Surgical prophylaxis only.

AHA, acquired hemophilia A; anti-FVIII, anti-FVIII activity; aPTT, activated partial thromboplastin time; BU, Bethesda units; CS, corticosteroids; CYC, cyclophosphamide; FK, tacrolimus; FVIII, coagulation factor VIII; Ig, immunoglobulin; IgG4-RD, IgG4-related disease; IS, immunosuppressants; RTX, rituximab.

**Figure 2:**
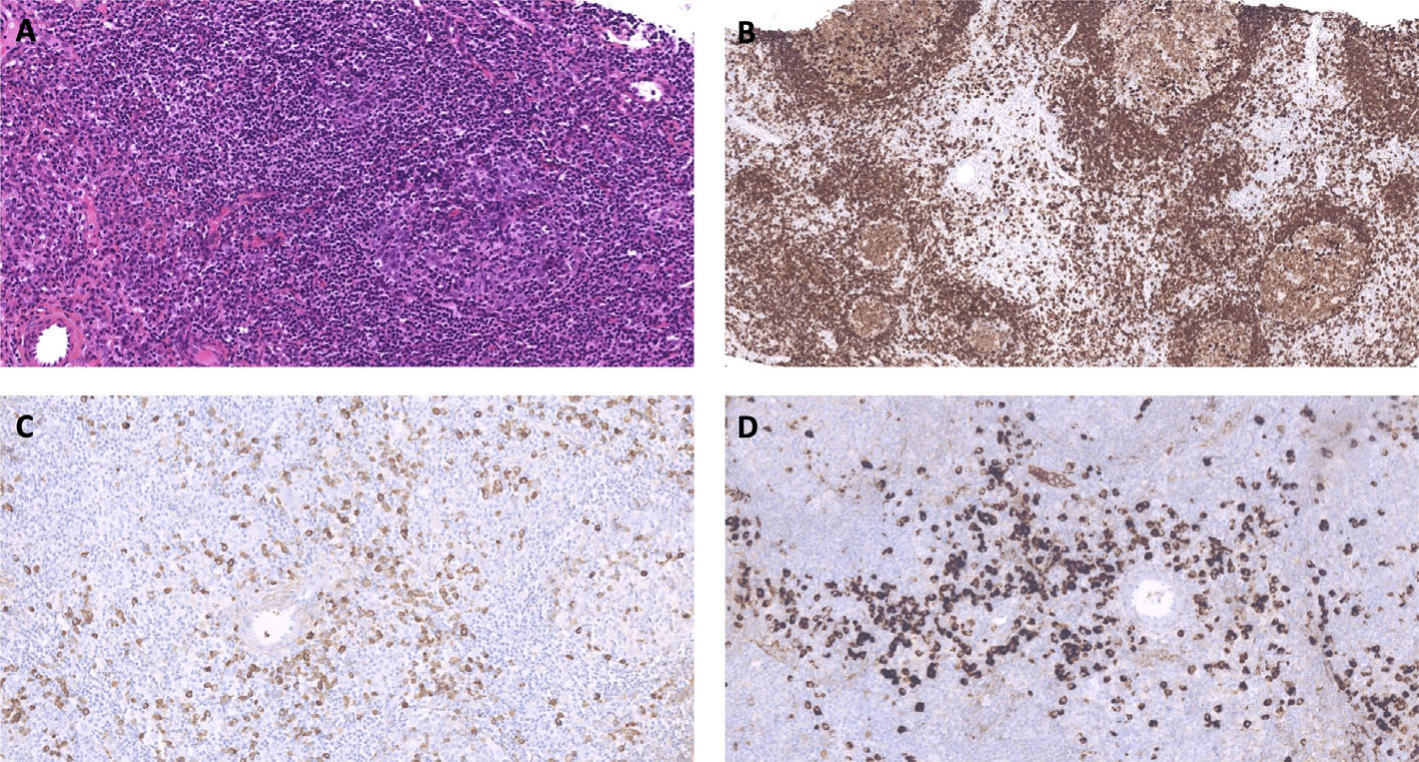
Representative images of the axillary lymph node biopsy histological examination. **(A)** Lymph node showing reactive follicular hyperplasia. The reactive follicle comprises germinal center surrounded by a thin mantle zone. The interfollicular area contains numbers of mature plasma cells. Hematoxylin and Eosin staining x400. **(B)** Lymph node showing reactive follicular hyperplasia and mature plasma cells. CD79a staining. **(C**, **D)** IgG **(C)** and IgG4 **(D)** staining. Numerous IgG4^+^ cells are present between follicles. The IgG4^+^/IgG^+^ cell proportion is over 40%, with more than 200 IgG4^+^ cells per high power field.

The axillary lymph node biopsy was finally performed under prophylactic infusions of bypassing agents (factor eight inhibitor bypassing activity [FEIBA]). Histological examination revealed benign follicular hyperplasia with elevated IgG4/IgG ratio and no evidence of lymphoma (**Figure 2**). Further laboratory tests showed elevated serum IgG4 levels (**Table 1** and **Figure 1**) with other IgG subclasses within normal range. The patient was thus diagnosed with parotid and lymph node IgG4-RD according to the 2019 ACR/EULAR classification criteria (7).

Overall, our patient was considered to have IgG4-RD-associated AHA and was started on oral prednisone 1mg/kg/day for both diseases. This led to a regression of parotid and lymph node enlargement, a decrease in serum IgG4 levels, a progressive correction of FVIII and aPTT values, and a suppression of anti-FVIII plasma activity (**Table 1** and **Figure 1**). Prednisone was then slowly tapered and discontinued after 9 months.

Nine months after treatment withdrawal, the patient relapsed. This was characterized by a new increase in IgG4 serum levels without organ involvement; and by a decrease in FVIII plasma levels with elevated anti-FVIII autoantibodies but without bleeding (**Table 1** and **Figure 1**). The patient was started on a new course of oral corticosteroids associated with 2 infusions of rituximab 1g, which led to a sustained remission (total follow-up of 3 years after diagnosis).

### Determination of Anti-FVIII IgG Subclass

As this patient presented with an unusual association of rare diseases, we wondered whether there was an immunopathogenic link between the 2 conditions. Our first hypothesis was that the anti-FVIII autoantibodies could be directly produced by the proliferating IgG4^+^ plasma cells as a result of broken tolerance to endogenous FVIII. To test this assumption, we tried to determine the subclass of the anti-FVIII IgG, both in our patient and in a control group of 11 patients diagnosed with AHA but with no evidence of IgG4-RD.

FVIII inhibitor was mostly of the IgG4 subclass, with an anti-FVIII IgG4/IgG1 ratio of 42 at diagnosis and 268 at relapse in our patient with IgG4-RD-associated AHA (**Table 2**). This ratio seemed lower in the AHA-only control group (median [min-max]: 10 [0,2->617]); however, some controls had elevated anti-FVIII IgG4/IgG1 ratios in ranges similar to that of our patient at diagnosis (AHA#1, #2, and #8: 56, 41, and 37 vs. 42, respectively) and at relapse (AHA#9: >617 vs. 268). Overall, these results suggest a preferential production of anti-FVIII antibodies of the IgG4 subclass in our patient, although this is also observed in non-IgG4-RD-associated AHA.

**Table 2:** Hemostatic and immunological explorations in our patient and in several control groups (AHA-only, IgG4-RD-only and healthy controls).

	Sex	Age	aPTT(s)	FVIII(%)	Anti-FVIII activity(BU/ml)	Total IgG4(g/l)	Total IgG1(g/l)	Anti-FVIII IgG(µg/ml)	Anti-FVIII IgG4(µg/ml)	Anti-FVIII IgG1(µg/ml)	Anti-FVIII IgG4/IgG1	Anti-FVIII IgG4/total IgG4 (x1000)
**OUR PATIENT**												
At diagnosis	M	75	72	1	27	**11.70**	7.04	67.43	31.49	0.75	**42**	**2.7**
At relapse		77	65	2	12	**18.22**	7.68	23.94	18.77	0.07	**268**	**1.0**
**AHA-ONLY CONTROLS**												
AHA #1	F	93	85	<10	8.9	0.71	4.86	129.8	63.88	1.15	**56**	**90.0**
AHA #2	M	76	48	15	1.7	0.30	4.37	24.67	15.0	0.37	**41**	**49.8**
AHA #3	M	72	77	<1	3.7	0.32	2.99	28.54	14.66	1.4	**10**	**46.0**
AHA #4	M	72	58	8,7	2	0.05	4.91	24.37	0.58	1.31	**0.4**	**11.6**
AHA #5	M	59	63	42	1.3	0.48	4.44	13.08	2.09	2.08	**1.0**	**4.4**
AHA #6	F	88	107	<1	6	0.92	6.88	26.11	10.35	2.39	**4.3**	**11.3**
AHA #7	F	81	86	1	6	0.21	4.34	99.95	36.96	4.54	**8.1**	**178.6**
AHA #8	F	87	66	2	42	0.39	4.81	65.49	34.88	0.95	**37**	**90.1**
AHA #9	M	86	49	6	1.5	0.57	2.57	4.58	1.85	<0.003	**>617**	**3.2**
AHA #10	F	63	74	2	2	0.17	2.82	1.01	0.10	0.49	**0.2**	**0.6**
AHA #11	F	87	105	1	8	0.28	3.98	9.19	6.66	0.42	**16**	**23.7**
***Median*** ***(min-max)***									***10,35 (0,10–63,88)***	***1,15 (<0.003–4,54)***	***10*** ***(0,2->617)***	***24*** ***(0,57-179)***
**IGG4-RD-ONLY CONTROLS**												
IgG4-RD #1	M	70	29			1.96	4.07	0.17	0.12	<0.003		**0.059**
IgG4-RD #2	M	79	34			18.27	9.97	1.00	0.43	<0.003		**0.024**
IgG4-RD #3	M	52	31			2.32	6.12	0.03	0.05	<0.003		**0.020**
IgG4-RD #4	M	69	40			2.01	13.29	0.11	0.05	0.01		**0.027**
IgG4-RD #5	F	67	27			4.75	5.60	0.07	0.08	<0.003		**0.017**
IgG4-RD #6	M	64	30			19.31	15.94	0.97	0.62	0.04		**0.032**
IgG4-RD #7	M	60	30			4.02	13.05	0.11	0.14	0.09		**0.035**
IgG4-RD #8	M	75	26			2.85	8.82	0.10	0.07	<0.003		**0.025**
***Median*** ***(min-max)***									***0,10 (0.05-0,62)***	***<0.003 (<0.003-0.090)***		***0.03*** ***(0.02-0.06)***
**HEALTHY CONTROLS**												
HC#1	F	63		72	0	0.475	5.913	0.08	<0.001	<0.003		**<0.002**
HC#2	M	62		118	0	0.037	4.742	0.36	<0.001	<0.003		**<0.027**
HC#3	M	50		131	0	0.034	7.310	0.07	<0.001	<0.003		**<0.030**
HC#4	M	69		141	0	0.452	6.103	0.05	0.05	<0.003		**0,118**
HC#5	M	55		144	0	0.542	3.990	0.04	0.04	<0.003		**0.070**
HC#6	M	65		159	0	0.346	4.870	0.02	<0.001	<0.003		**<0.003**
HC#7	F	55		172	0	0.054	5.778	0.07	<0.001	<0.003		**<0.019**
HC#8	F	60		160	0	0.337	4.477	0.04	<0.001	<0.003		**<0.003**
HC#9	F	59		188	0	0.595	6.011	0.03	<0.001	<0.003		**<0.002**
HC#10	M	52		192	0	0.216	5.883	0.04	<0.001	<0.003		**<0.005**
HC#11	F	52		217	0	0.942	8.035	0.05	<0.001	<0.003		**<0.001**
***Median*** ***(min-max)***									***0.001 (<0.001–0.053)***	***<0.003 (<0.003–<0.003)***		***0.005 (<0.001–0.118)***

AHA, acquired hemophilia A; anti-FVIII, anti-FVIII activity; aPTT, activated partial thromboplastin time; BU, Bethesda units; F, female; FVIII, coagulation factor VIII; HC, healthy controls; Ig, immunoglobulin; IgG4-RD, IgG4-related disease; M, male; SD, standard deviation. We used bold and bold-italicized characters in order to make the most important data more visible.

### Assessment of the Anti-FVIII IgG4/Total IgG4 Ratio

**Figure 3:**
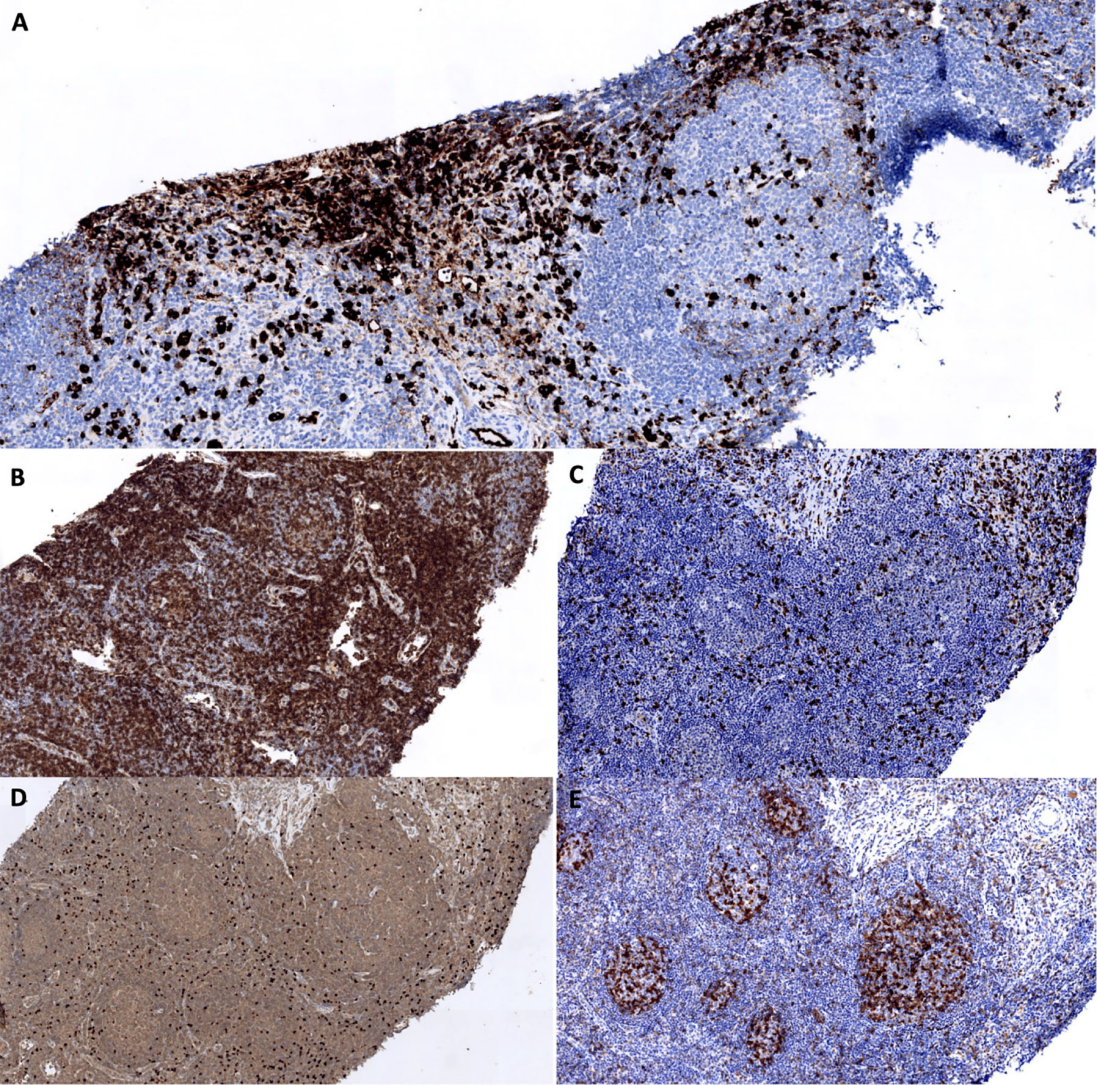
Assessment of TFH and Treg infiltrates in the lymph node biopsy. **(A)** IgG4 staining. The interfollicular area contains numbers of IgG4^+^ cells. **(B–E)** CD4 **(B)**, CD8 **(C)**, FOXP3 **(D)** and PD1 **(E)** staining. Expansion of CD4^+^ PD1^+^ cells within follicles. Numerous CD4^+^ FOXP3^+^ cells are present in interfollicular areas.

Our second hypothesis was that an undetectable anti-FVIII activity exists physiologically within the normal human IgG4 repertoire, and that the polyclonal expansion of this repertoire observed during IgG4-RD increases the anti-FVIII fraction above detection thresholds (**Figure 3**). To test this hypothesis, we assessed the anti-FVIII IgG4/total IgG4 ratio in our patient, as well as in several control groups: 11 AHA patients without IgG4-RD, 8 IgG4-RD patients without AHA, and 11 healthy controls (**Table 2**).

In the AHA without IgG4-RD control group, the median anti-FVIII IgG4/total IgG4 ratio was 2.4 10^-2^ (5.7 10^-4^-1.79 10^-1^). In this setting, the anti-FVIII antibody production could be considered to be the result of broken tolerance to endogenous FVIII and oligoclonal expansion of a pathogenic plasma cell contingent (12).

In the IgG4-RD without AHA control group, the median anti-FVIII IgG4/total IgG4 ratio was 3.0 10^-5^ (2.0 10^-5^-6.0 10^-5^). In this setting, the detected anti-FVIII activity is most likely the result of a bystander antibody production due to polyclonal IgG4^+^ plasma cell proliferation.

In the healthy control group, most subjects had no detectable anti-FVIII IgG4, except for HC#4 and #5 who had anti-FVIII IgG4/total IgG4 ratios in ranges similar to that of the IgG4-RD-only patients (1.2 10^-4^ and 7.0 10^-5^, respectively). This might comfort the hypothesis of a usually undetectable, non-specific physiological anti-FVIII activity, that increases above detection levels during IgG4-RD.

In our patient, anti-FVIII IgG4 represented only a small proportion of the total serum IgG4, with a ratio of 2.7 10^-3^ at diagnosis and 1.0 10^-3^ at relapse. These values seemed lower than that measured in the AHA-only group and higher than that in the IgG4-RD-only group. Overall, these results suggest that a combination of both pathogenic mechanisms (i.e., broken tolerance to FVIII and bystander polyclonal IgG4^+^ plasma cell proliferation) may be at work in our patient.

### Assessment of TFH and Treg Infiltrates in Lymph Nodes

In order to better study whether tolerance to FVIII was broken in our patient, we assessed the level of TFH and Treg infiltrations in the lymph node biopsy. We observed a TFH expansion within hyperplastic follicles, and a Treg infiltration in interfollicular areas close to IgG4^+^ plasma cells (**Figure 3**). These findings did not seem particularly different from those observed in control patients with simple follicular hyperplasia.

## Discussion

We report here a case of AHA associated with IgG4-RD, an unusual combination of 2 rare immunological diseases, with a favorable outcome under corticosteroids and rituximab. We postulated that there could be an immunopathogenic relationship between the 2 conditions, and explored 2 pathophysiological hypotheses: (1) broken tolerance to endogenous FVIII induced an antigen-driven oligoclonal proliferation of IgG4^+^ plasma cells and the production of anti-FVIII autoantibodies; and (2) a polyclonal proliferation of IgG4^+^ plasma cells occurred that increased a bystander anti-FVIII activity that exists physiologically below detection levels.

### Review of the Literature

In our patient, both diseases were diagnosed according to standard-of-care recommendations: the patient met the clinical, serological and histological items of the ACR/EULAR 2019 classification criteria for IgG4-RD (7); and the presence of a FVIII inhibitor demonstrated by a gold-standard technique (Bethesda assay) associated with low FVIII titers confirmed AHA diagnosis (3). It is however interesting to note that the presenting features of AHA are quite unusual: indeed, asymptomatic AHA is rare and has been reported in only 6.6% of the cases.

So far, the co-occurrence of AHA and IgG4-RD has only been reported in 3 published cases (4–6) (**Table 1**). In all cases, patients presented with suggestive organ involvements (notably auto-immune pancreatitis, sclerosing cholangitis, salivary gland enlargement, pulmonary nodules and interstitial lung disease), elevated serum IgG4 levels and cardinal histological features that met the 2019 ACR/EULAR classification criteria for IgG4-RD (7). Following disease onset, they were referred for hemorrhagic events (mostly subdermal and muscular hematomas) associated with prolonged aPTT, low FVIII level and positive anti-FVIII Ig screening test, which led to the diagnosis of AHA. All patients were managed with a course of high-dose corticosteroids, associated with immunosuppressants in 1 case (4). By-passing agent therapy was frequently needed. Relapse occurred in 1 case and was managed by a change of immunosuppressing strategy (4). Outcome was favorable in all cases.

Another article reported the observation of a patient presenting with AHA and high serum IgG4 levels (13). However, in this case, the diagnosis of IgG4-RD seems uncertain: aside from elevated serum IgG4 levels, the authors mentioned no organ involvement and no histological documentation suggestive of the disease; furthermore, the patient displayed markedly increased eosinophil counts and TCR clonality, which is compatible with a diagnosis of hypereosinophilic syndrome (lymphocytic-variant).

### AHA and IgG4-RD: An Immunopathogenic Link?

As to investigate the possibility of a pathophysiological relationship between AHA and IgG4-RD in our patient, we explore 2 distinct hypotheses.

As a first hypothesis, we wondered if the anti-FVIII autoantibody production was the result of an oligoclonal proliferation of IgG4^+^ plasma cells driven by broken tolerance to endogenous FVIII. We found that anti-FVIII IgG were indeed mostly of the IgG4 subclass in our patient. Interestingly, this was also observed in another IgG4-RD-associated AHA case (4). In the latter patient, Narazaki et al. showed that the FVIII inhibitor bound to the 200-kDa heavy chain and the 80-kDa light chain of non-activated FVIII, and to the 50- and 40-kDa fragments of the heavy chain, and a 72-kDa fragment of the light chain of activated FVIII (4). However, we also found a preferential secretion of anti-FVIII IgG4 in non-IgG4-RD AHA controls, which suggests that this phenomenon is not specific to IgG4-RD-associated AHA. This is concordant with previous publications which demonstrate that anti-FVIII IgG are usually IgG4 or IgG1 autoantibodies in AHA (14).

The precise role of IgG4 antibodies in the pathophysiology of IgG4-RD remains unclear; and their antigen specificity is controversial (2). Nevertheless, autoreactive IgG4 have been previously documented in this disease: notably, Saeki et al. reported the case of a patient diagnosed with IgG4-RD and thrombotic thrombocytopenic purpura, who displayed anti-ADAMTS-13 antibodies of the IgG4 subclass (15).

**Figure 4:**
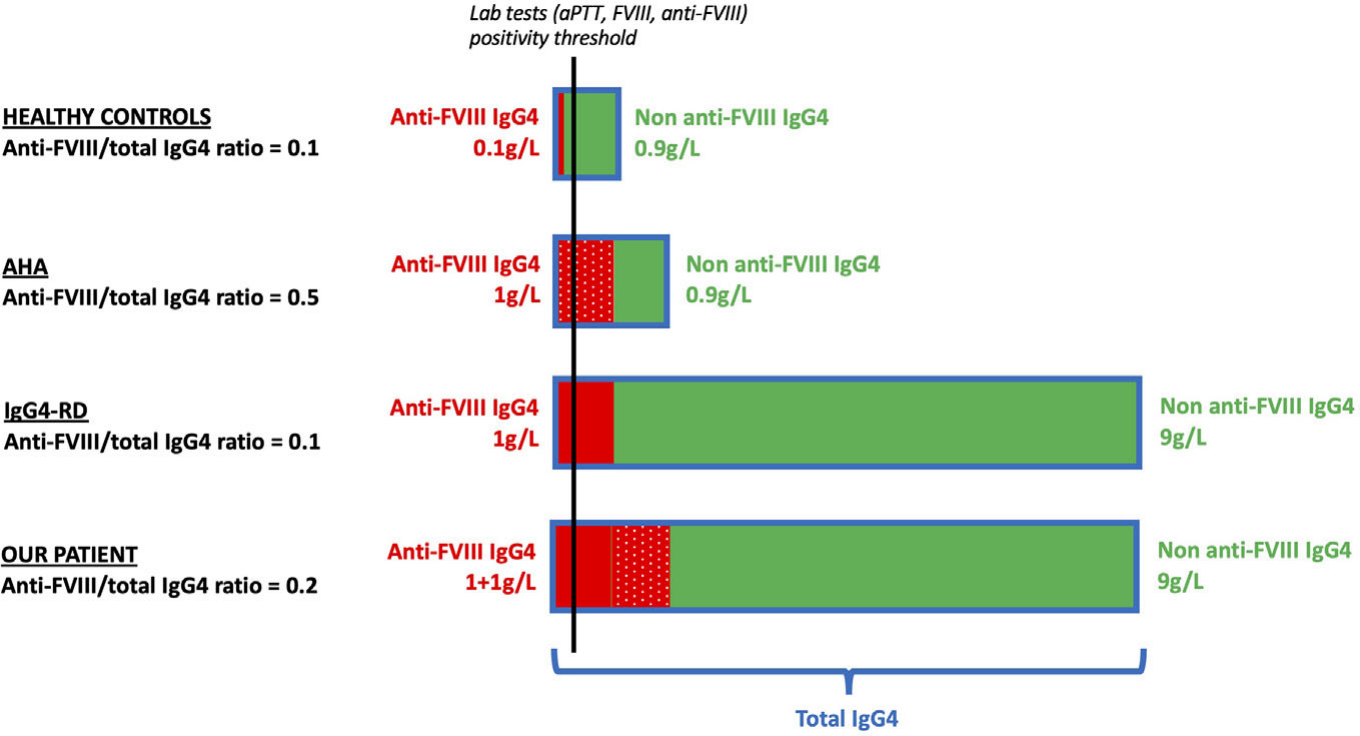
Schematic representation of the putative mechanisms responsible for anti-FVIII antibody production in healthy controls (first row), AHA patients (second row), IgG4-RD patients (third row), and in our patient (fourth row). We hypothesized the ratios of anti-FVIII IgG4/total IgG4 in four populations: *First row: healthy controls.* A small fraction of the IgG4 repertoire has anti-FVIII activity (plain red) compared to non-anti-FVIII IgG4 (green). The anti-FVIII IgG4/total IgG4 ratio is 0.1. *Second row: AHA.* The anti-FVIII IgG4 fraction (dotted red) is produced by an antigen-driven oligoclonal expansion of a small contingent of pathogenic plasma cells. The rest of the IgG4 repertoire (green) remains similar to healthy controls. The anti-FVIII IgG4/total IgG4 ratio is 0.5. *Third row: IgG4-RD.* The anti-FVIII IgG4 fraction (plain red) is the result of an overall expansion of the IgG4 repertoire. As the anti-FVIII (plain red) and non-anti-FVIII (green) IgG4 fractions are both increased in similar proportion to healthy controls, the anti-FVIII IgG4/total IgG4 ratio is 0.1 (identical to healthy controls and lower than AHA). *Fourth row: our patient.* The anti-FVIII is a combination of both previous mechanisms: bystander production by polyclonal proliferation of IgG4^+^ plasma cells (plain red) and antigen-driven production due to broken tolerance to FVIII leading to (dotted red). The non-anti-FVIII (green) is increased in similar proportion to IgG4-RD patients. The anti-FVIII IgG4/total IgG4 ratio is 0.2 (higher than IgG4-RD and lower than AHA). In order to simplify the explanation, qualitative values are displayed. AHA, acquired hemophilia A; anti-FVIII, anti-FVIII activity; aPTT, activated partial thromboplastin time; FVIII, coagulation factor VIII; HC, healthy controls; Ig, immunoglobulin; IgG4-RD, IgG4-related disease.

As a second hypothesis, we considered whether the anti-FVIII activity could be the result of a bystander autoantibody production due to polyclonal IgG4^+^ plasma cell proliferation. To test this hypothesis, we used the anti-FVIII IgG4/total IgG4 ratio as a surrogate to assess which proportion the anti-FVIII IgG4^+^ plasma cells represented within the whole IgG4-producing cells. Unsurprisingly, we found that this ratio was higher in AHA-only controls (an oligoclonal setting in which only anti-FVIII IgG4^+^ plasma cells proliferate) than in IgG4-RD-only controls (a polyclonal setting in which all IgG4^+^ plasma cells proliferate equally) (**Figure 4**). Our patient had intermediate ratio values, which could plead for a combination of both mechanisms. The study of T cell responses (as assessed by TFH and Treg immunostaining on lymph node sections) unfortunately did not provide further insight into this issue.

## Conclusion

We report herein the observation of a patient with IgG4-RD and AHA, an unusual combination of two rare diseases, and show the FVIII inhibitor was mostly an IgG4 autoantibody. Although no definitive conclusion can be drawn from our work, we hypothesize that the anti-FVIII autoantibody production could be the result of both broken tolerance to FVIII and bystander polyclonal IgG4^+^ plasma cell proliferation.

## Data Availability Statement

The original contributions presented in the study are included in the article/supplementary material. Further inquiries can be directed to the corresponding author.

## Ethics Statement

Ethical review and approval was not required for the study on human participants in accordance with the local legislation and institutional requirements. Written informed consent for participation was not required for this study in accordance with the national legislation and the institutional requirements. Written informed consent was obtained from the individual(s) for the publication of any potentially identifiable images or data included in this article.

## Author Contributions

All individuals listed as authors met the ICMJE guidelines for determining authorship. SSa, EJ, BL, DL, SL-D, and LT contributed to the conception and design of the study. EJ, BL, JR, SDe and SL-D performed the experiments and acquired the data. SS constituted the database. SS wrote the first draft of the manuscript. EJ, BL, DL, SL-D, and LT made major contributions to the manuscript. RD performed the histological work-up, provided the iconography for **Figures 2** and **3**, and drafted its legend. SDu and TG provided their expertise on T cell responses and helped interprete the results of TFH and Treg immunostainings. BC, ÉH, and P-YH provided their IgG4-RD expertise. CP and SSu provided their AHA expertise. All authors read and approved the submitted version.

## Conflict of Interest

The authors declare that the research was conducted in the absence of any commercial or financial relationships that could be construed as a potential conflict of interest.
